# High-Performance Near-Infrared Photodetector Based on PbS Colloidal Quantum Dots/ZnO-Nanowires Hybrid Nanostructures

**DOI:** 10.3390/s23042254

**Published:** 2023-02-17

**Authors:** Hefu Zhong, Libin Tang, Pin Tian, Lijing Yu, Wenbin Zuo, Kar Seng Teng

**Affiliations:** 1 School of Materials and Energy, Yunnan University, Kunming 650500, China; 2 Kunming Institute of Physics, Kunming 650223, China; 3 Yunnan Key Laboratory of Advanced Photoelectric Materials and Devices, Kunming 650223, China; 4 Department of Electronic and Electrical Engineering, Swansea University, Bay Campus, Fabian Way, Swansea SA1 8EN, UK

**Keywords:** ZnO NWs, PbS, CQDs, infrared detector

## Abstract

Quantum dots have found significant applications in photoelectric detectors due to their unique electronic and optical properties, such as tunable bandgap. Recently, colloidal quantum dots (CQDs) have attracted much interest because of the ease of controlling the dot size and low production cost. In this paper, a high-performance ZnO/PbS heterojunction photodetector was fabricated by spin-coating PbS CQDs onto the surface of a hydrothermally grown vertical array of ZnO nanowires (NWs) on an indium tin oxide (ITO) substrate. Under 940 nm near-infrared light illumination, the device demonstrated a responsivity and detectivity of ~3.9 × 10^4^ A/W and ~9.4 × 10^13^ Jones, respectively. The excellent performances and low cost of this nanocomposite-based photodetector show that it has the potential for widespread applications ranging from medical diagnosis to environmental monitoring.

## 1. Introduction

Photodetectors have very extensive and important military and civilian applications, such as missile early warning systems [1], medical diagnosis [2,3,4,5] and environmental monitoring [6,7,8,9]. Among the photosensitive materials, PbS CQDs have been widely used in the design and development of photodetectors due to their tunable size, controllable bandgap and low-cost solution processing technique [10,11]. Thanks to these characteristics, a large number of researchers have conducted synthesis and application research on PbS CQDs. At present, the main preparation technologies include hydrothermal and electrochemical deposition, and these materials have been widely used in the fields of solar cells, biosensors and photocatalysis, etc. [12]. In addition, through the exchange and modification of PbS CQD surface ligands, it is easy to obtain good-performance QD hybrid nanostructures [13,14]. However, the carrier mobility of PbS CQDs is relatively low, which is known to limit device performance [15]. A common approach is to combine PbS CQDs with materials having high carrier mobility to improve device performance [15,16].

ZnO is a direct, wide bandgap (3.37 eV) semiconductor with large exciton binding energy (60 meV) [17]. The transparent conductive oxide was previously combined with PbS CQDs in the fabrication of photodetectors [18,19]. However, various trap states are prone to exist at the PbS/ZnO interface, which could lead to the recombination of carriers at the interface, hence limiting the performance of PbS/ZnO heterojunction optoelectronic devices [20]. The use of ZnO nanowires to improve device performance has attracted great interest [21,22]. The one-dimensional nanowires offer rapid electron transport and direct conduction paths, which could reduce the recombination of photogenerated carriers and greatly enhance the photoelectric response of the photodetector [23].

Recently, there are reports on the fabrication of photodetectors based on ZnO NWs/PbS CQDs hybrid nanostructures. For example, Deep Chandra Upadhyay et al. [24] prepared a ZnO nanorod/PCDTBT:PCBM:PbS QDs/MoOx heterojunction using FTO (4.4 eV) as a substrate to grow ZnO nanorods (NRs) by a hydrothermal method. The device exhibited a detectivity of 5.82 × 10^11^ Jones in the near-infrared band. Nitumoni Deka et al. [25] prepared a ZnO NRs/PbS/RGO heterojunction photodetector by depositing PbS QDs onto ZnO NRs using chemical bath deposition on an FTO substrate, followed by spin-coating a layer of reduced graphene oxide (RGO). The device demonstrated a detectivity of ~10^4^ Jones in the near-infrared band. The relatively poor detectivity of the above devices may be due to the low work function of FTO.

Although the dark current of photodetector hybrids of ZnO NWs and PbS CQDs may be large, the high specific surface area (larger than NRs) [26,27] and good charge transfer characteristics of ZnO NWs make it possible to prepare a high-performance ZnO NWs/PbS CQDs hybrid near-infrared detector. In this work, we made a completely new device structure. An ITO (4.7 eV) [28] substrate was used and investigated in the fabrication of a ZnO NWs/PbS CQDs hybrid nanostructure-based photodetector to enhance device performance. The ZnO NWs were used as the hole transport layer (HTL) and PbS CQDs were used as the infrared absorption layer [29]. The structure and morphology of PbS CQDs and ZnO NWs were characterized, and the photoelectric properties of the infrared detectors were also studied. 

## 2. Experiment

### 2.1. Chemical Reagents

The chemical reagents used in this experiment were: zinc acetate (Purity: AR, Xi Long Scientific Co., Ltd., Guangdong, China), hexamethylene tetramine (Purity: AR, Xi Long Scientific Co., Ltd.), ethanolamine (Purity: AR, Tianjin Feng Chuan Chemical Reagent Co., Ltd., Tianjin, China), methanol (Purity: AR, Suzhou Crystal Clear Electronic Material Co., Ltd., Suzhou, China), octane (Purity: AR, Tianjin Zhi Yuan Chemical Reagent Co., Ltd., Tianjin, China), ammonia solution (Purity: AR, Tianjin Feng Chuan Chemical Reagent Co., Ltd.) and 2-methoxyethanol (Purity: AR, China National Medicines Corporation Co., Ltd., Beijing, China). All chemical reagents were used as purchased without further purification.

### 2.2. Device Fabrication

ITO quartz substrate (30 Ω•sq^−1^) was cleaned and heated for 30 min in a mixture of methanol, ammoniac and deionized water in a 1:1:2 ratio, and then rinsed several times with deionized (DI) water. A layer of ZnO seed layer was coated onto the ITO substrate. The solution of ZnO seed layer was prepared by mixing 1.5 g zinc acetate, 20 mL 2-methoxyethanol and 450 μL ethanolamine. This was then heated and stirred using a magnetron agitator at 80 °C for 30 min, and the solution was cooled to room temperature prior to use. The ITO substrate coated with ZnO seed layer was then annealed at 320 °C for 1 h. A ZnO NWs array was grown by a hydrothermal method in a mixture consisting of zinc acetate (0.02 M), hexamethylene tetramine (0.02 M) and ammonia solution (28 wt%) mixed with 45 mL DI water at room temperature, similar to a previous approach [30]. This was followed by the addition of the ITO substrate coated with ZnO seed layer into the mixture and heated at 90 °C for 6 h, then rinsed with DI water several times and finally blow-dried with nitrogen. The concentration of the PbS CQDs (Purchased from Huazhong University of Science and Technology) was 30 mg/mL and the solvent was octane. 

### 2.3. Structural and Device Characterization

The morphology and structure of the ZnO NWs were studied using a Nova Nano SEM 450 spectrometer (SEM). The ZnO NWs and PbS CQDs were characterized both by X-ray diffraction (XRD) using a Rigaku D/Max-23 diffractometer at room temperature and transmission electron microscopy (TEM) using a Tecnai G2 F30 S-Twin microscope. A Keithley 2400 source meter was used to measure the current density-voltage (*J-V*) characteristics of the device.

## 3. Results

**Figure 1:**
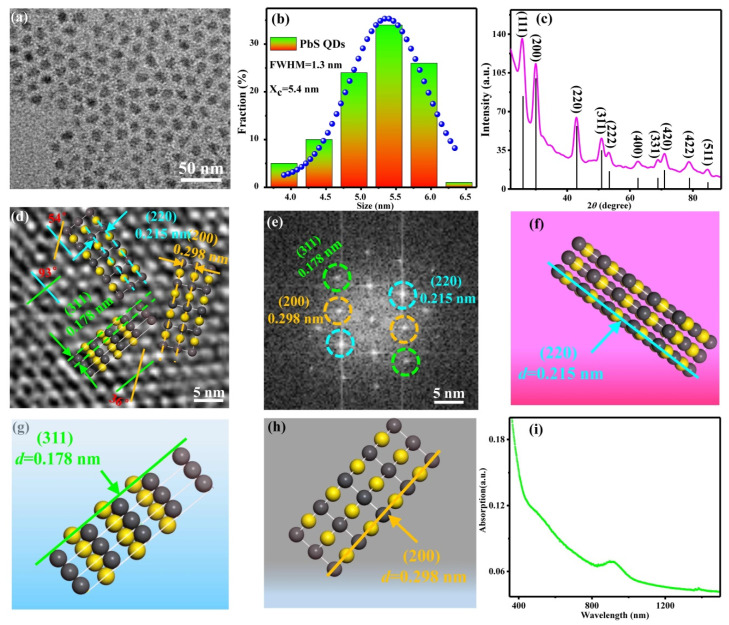
Structural characterization of PbS CQDs. (**a**) TEM image. (**b**) Histogram illustrating the particle size distribution. (**c**) XRD pattern. (**d**) HRTEM image. (**e**) FFT spectra. (**f**–**h**) Crystal structures of PbS CQDs with (220), (311) and (200) faces, respectively. (**i**) Optical absorption spectrum.

Figure 1a shows a TEM image of the PbS CQDs. The PbS CQDs exhibited uniform size and good dispersion. Figure 1b shows the particle size distribution of the PbS CQDs. The histogram revealed the average size of the PbS CQDs was ~5.5 nm with a full width at half maximum (FWHM) of 1.3 nm. Figure 1c,d show the XRD pattern and high-resolution TEM (HRTEM) image of the PbS CQDs, respectively. The observed crystal faces of (220), (311) and (200) in Figure 1d were in good agreement with the peaks in the XRD spectrum shown in Figure 1c. The angles between crystal faces (200) and (220), (311) and (220) and (311) and (200) were 54°, 93° and 36°, respectively. Furthermore, lattice fringes can be observed in the HRTEM image, suggesting that the PbS CQDs exhibited good crystal structure. The fast Fourier transform (FFT) spectra of the PbS CQDs also indicated good crystallinity as shown in Figure 1e. Schematic diagrams illustrating the crystal structures of PbS CQDs with (220), (311) and (200) faces are depicted in Figure 1f, g and h, respectively. As shown, the interplanar spacings of the (220), (311) and (200) faces were 0.215, 0.178 and 0.298 nm, respectively. Figure 1i shows the optical absorption spectrum of the PbS CQDs, which revealed an absorption peak at 900 nm, indicating that they are suitable for near-infrared applications. 

**Figure 2:**
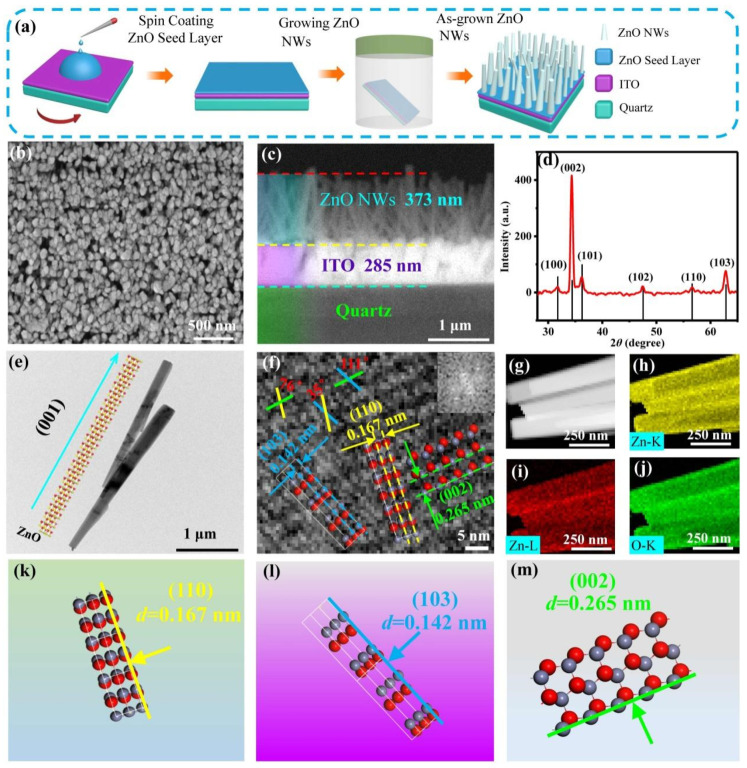
Preparation and structural characterization of ZnO NWs. (**a**) Schematic diagram illustrating the preparation process of ZnO NWs grown by hydrothermal method. (**b**) Top view SEM image. (**c**) Cross-sectional SEM image. (**d**) XRD spectrum. (**e**) TEM image. (**f**) HRTEM image of individual ZnO NWs (Inset: FFT spectrum of ZnO NWs). (**g**) STEM image of ZnO NWs. (**h**–**j**) Elemental mapping of Zn and O. (**k**–**m**) Schematic diagrams illustrating the crystal structures of ZnO NWs with (110), (103) and (002) faces.

Figure 2a illustrates the preparation process of the ZnO NWs grown by a hydrothermal method. Figure 2b shows a top-view SEM image of the as-grown ZnO NWs, which exhibited relatively uniform growth. Figure 2c shows a cross-sectional SEM image of the vertical array of the ZnO NWs with a height of 373 nm grown on the ITO substrate. Figure 2d shows the XRD pattern of the ZnO NWs. The diffraction peak of (002) was the largest among the (100), (101), (102), (110) and (103) faces, indicating the preferential growth of the nanowire along the (002) orientation. Figure 2e shows a TEM image of ZnO NWs grown along the direction perpendicular to the (001) crystal plane (i.e., c-axis direction) [30]. The inset shows a schematic diagram of the crystal structure of ZnO NWs grown perpendicular to the (001) crystal plane. Figure 2f shows the HRTEM image of ZnO NWs with the FFT spectrum shown as an inset. The image revealed lattice fringes at the ZnO NWs, indicating that they exhibited good crystallinity. The angles between the (110) and (002), (110) and (103) and (103) and (002) crystal planes were 76°, 35° and 111°, respectively. Figure 2g shows a scanning TEM (STEM) image of ZnO NWs, in which elemental mapping analysis was performed. Figure 2h–j show the STEM-energy dispersive X-ray (STEM-EDX) elemental mapping of Zn and O. The mapping revealed only Zn and O elements, indicating the high purity of the as-grown ZnO NWs. Figure 2k–m show schematic diagrams of the crystal structures of ZnO NWs with (110), (103) and (002) faces, respectively. The interplanar spacings of the (110), (103) and (002) faces were 0.167, 0.142 and 0.265 nm, respectively. The good crystal quality of the as-grown ZnO NWs would be beneficial to the direct conduction of photogenerated carriers.

**Figure 3:**
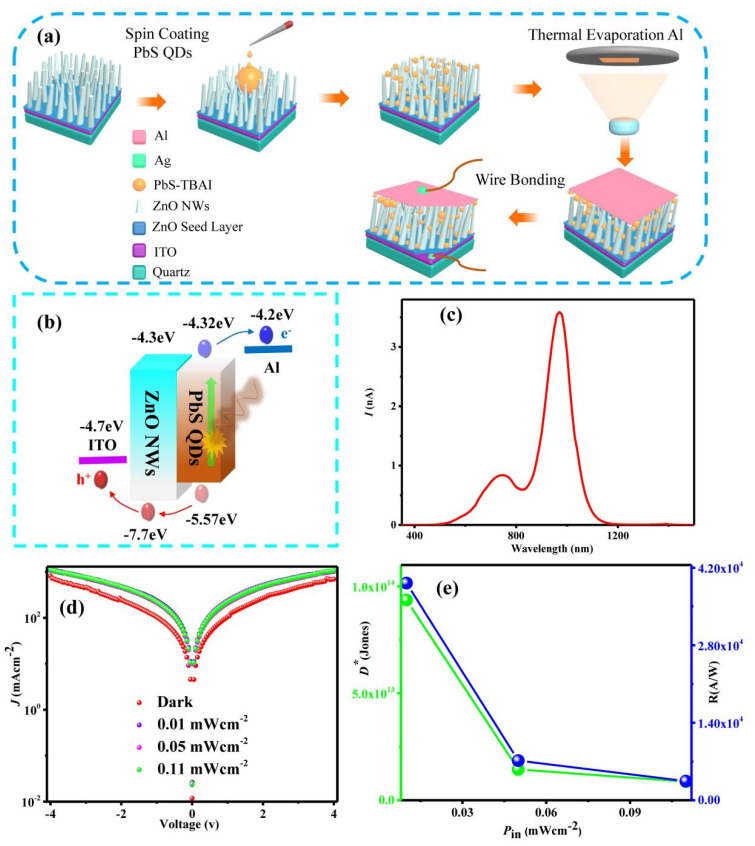
Fabrication and performance of the photodetector based on ZnO NWs/PbS CQDs hybrid nanostructures. (**a**) Schematic diagram illustrating the fabrication process of the photodetector. (**b**)The energy band diagram of the photodetector. (**c**) The photocurrent spectrum of the photodetector. (**d**) *J-V* characteristics of the photodetector at different infrared power densities (λ = 940 nm). (**e**) Responsivity and detectivity plots of the photodetector.

Figure 3a depicts the fabrication process of the infrared photodetector based on ZnO NWs/PbS CQDs hybrid nanostructures. PbS CQDs (30 mg/mL) were spin-coated onto ZnO NWs, followed by thermal evaporation of Al to form the upper electrode. Finally, a gold wire was connected to the Al electrode using silver conductive glue. Figure 3b is the energy band diagram of the device [16,31]. We choose ZnO NWs as the HTL and PbS CQDs as the infrared light absorption layer. When light irradiated the infrared light absorption layer, electron-hole pairs were separated, electrons flowed from the infrared light absorption layer to the Al electrode, and holes flowed to the ITO through the HLT. Figure 3c is the photocurrent spectrum of the device. At 764 nm and 967 nm, a photogenerated current was found in the device. The photodetector was characterized under an illumination of 940 nm near-infrared light at different power densities (0.01, 0.05 and 0.11 mWcm^−2^). Figure 3d shows the *J-V* characteristics of the photodetector at different power densities. There was no significant change in the photocurrent probably due to the relatively large dark current. Figure 3e shows the responsivity (*R*) and detectivity (*D*^∗^) plots of the device. The maximum responsivity and detectivity were ~3.9 × 10^4^ A/W and ~9.4 × 10^13^ Jones, respectively. It is worth noting that the *R* and *D*^∗^ decreased with an increase in the power density of the near-infrared light, which suggested that the device was suitable for detecting weak near-infrared signals. The *R* [32] and *D*^∗^ [33] of the device were calculated using the following formula:

(1) $$R=I_{ph}/PS$$

(2) $$D^{*}=R/{\left(2qJ_{d}\right)}^{1/2}$$

where *I_ph_* = *I_light_* − *I_dark_*, *P* is the incident power density, *S* is the effective device area, *J_d_* is the dark current density and *q* is the coulomb charge of an electron. Table 1 provides a list of the performances of other photodetectors based on PbS CQDs heterojunctions. This shows that the device fabricated in this work exhibits a higher responsivity and detectivity than other devices. Due to the large specific surface and one-dimensional carrier transmission channel of ZnO NWs, the photosensitive area was increased and the mobility of photogenerated carriers was also improved, which greatly improved device performance.

## 4. Conclusions

A vertical array of ZnO NWs was grown using a hydrothermal method on an ITO substrate. An infrared photodetector based on ZnO NWs/PbS CQDs hybrid nanostructures was fabricated. The ZnO NWs served as the ETL and the PbS CQDs as the infrared absorption layer. ITO and Al were used as the lower and upper electrodes, respectively. Under 940 nm near-infrared light illumination, the *R* and *D*^∗^ of the device were ~ 3.9 × 10^4^ A/W and ~9.4 × 10^13^ Jones, respectively. The photodetector demonstrated excellent performance suitable for a wide range of applications, such as object identification, optical communication, weak-light detection, etc.

## Figures and Tables

**Table 1:** List of photodetectors based on PbS CQDs heterojunctions and their performances.

Device	Illumination (nm)	Responsivity (A/W)	Detectivity (Jones)	Ref.
FTO/ZnO nanorods arrays/PCBM/ PbS CQDs/PCDTBT/MoOx/Ag	860	7.22	5.82 × 10^11^	[24]
FTO/ZnO-nanorods/PbS/graphene oxide	400	0.25	8.3 × 10^4^	[25]
ITO/ZnO/PbS_0.4_Se_0.6_/Au	980	25.8	1.3 × 10^13^	[34]
ITO/NiO/PbS/ZnO/Al	1135	/	1.1 × 10^12^	[35]
ITO/ZnO/PbS-TBAI/PbS-EDT/Au	1125	/	3.2 × 10^11^	[36]
ITO/ZnO NWs/PbS CQDs/Al	940	3.9 × 10^4^	9.4 × 10^13^	This work

## Data Availability

Not applicable.
